# Leeds General Infirmary

**Published:** 1892-10-29

**Authors:** 


### The Institutional Workshop.

### WITHIN THE HOSPITALS.

### LEEDS GENERAL INFIRMARY.

By our Special Commissioner.

No visitor who has a knowledge of hospital administration can fail to be struck with the General Infirmary at Leeds. It has structural defects which proved difficult to deal with when it was first opened, but we understand from the resident medical officers that at the present time the Infirmary is free from the evils which at first rendered treatment somewhat difficult. Quite recently the new out-patients' department and new wards have been made ready for occupation, and are now in use. These wards are not so large as those in the main building, but are well proportioned, and their floors of polished oak blocks two feet square, grouted in cement, present a pleasant appearance, and offer solid resistance to heavy weights, so that there is practically no noise from the traffic. These floors are worthy the attention of hospital authorities and architects. They were supplied by Messrs. Ebner and Co., 150, Old Street, St. Luke's, and cost 12s. 6d. per yard.

We were a little sorry to see that although the baths and bath-rooms have rounded glazed brick edges to the floors, which enables them to be easily cleansed, and are excellent, the slop and other sinks are not of porcelain, nor are they, in our judgment, of sufficient size to render them as efficient as they ought to be in a great hospital. This last is no doubt a matter of detail, and could easily be remedied when funds are available. We mention it because it is essential that every architect who undertakes hospital construction should make himself master of details like this, and so save the committee's unnecessary expenditure, and at the same time secure that every appliance fixed shall be of the latest and most approved pattern for the special purposes to which it will be put. The old wards contain thirty-two beds. Each is placed in charge of a sister, who has under her a charge nurse and two probationers, a staff which is increased in the case of the heavier wards to five nurses in all, including the sister. When the number of bad cases in a particular ward is unduly large, extra nurses are put on. These wards are spoilt to a great extent, so far as appearance is concerned, by the massive beams and girders which cross the ceiling at intervals. These wards, however, are splendidly kept. Some of the pictures and furniture are unusually good, and the general effect as well as the results of treatment are satisfactory. In some of these large wards there are wooden bedsteads, a circumstance which might fill the minds of some hospital authorities with horror. We were assured, however, by one of the sisters who has been in charge of a ward containing thirty-two beds for fourteen years, that during the whole of that time she has had no trouble arising from these bedsteads, and that on no single occasion has it been necessary to remove any one of them from the wards for the purpose of cleansing. This evidence shows what can be accomplished by efficient nursing and sound administration, seeing that many of the patients come to the hospital in a filthy condition, and that unless they were dealt with promptly the wooden bedsteads must long ago have been discarded as unfit for hospital use.

#### The Progress of the Infirmary.

The General Infirmary at Leeds has made great strides since the present buildings were first opened. It now treats upwards of 5,000 in-patients (the number of beds in daily occupation being usually 300), in addition to 28,864 out-patients, besides administering a large maternity department, and a dispensary from which 4,351 home visits to patients were paid last year by the medical staff. It also controls the Ida Hospital, a sort of convalescent branch of the Infirmary, which contained thirty-nine beds in daily occupation during 1891. It is interesting to know that whereas the number of in-patients under treatment in 1850 was 1,657, in 1860 the in-patients had increased to 1,982; in 1870 to 2,548; in 1880 to 3,523; and in 1890 to 4,994. Between the years 1850 and 1869 the number of in-patients remained practically at a standstill, the average number being under 1,700. With the opening of the new infirmary in 1870 a steady growth in numbers began. This growth amounted to about 33 per cent. in the first ten years, a rate which has been maintained to the present time, as may be seen from the number of in-patients treated already given. The out-patients we shall deal with later.

The most important and largest item on the income side of the accounts is as it ought to be, annual subscriptions and work-people's contributions. In 1850 this item stood at £2,029, in 1860 it increased to £2,519, in 1870 to £4,405, in 1880 to £5,420, and in 1890 to £8,728. It will be seen from these figures that whereas the increase in the income from these two sources was about 25 per cent. only during the decade ending 1880, compared with an increase in the in-patients of 33 per cent., the growth of income in the last ten years has been so considerable as practically to reduce the balance between these two items. Thus the average percentage of increase in income at the present time is almost identical with the growth in the in-patients; any difference being in favour of the percentage of increase shown by the income, as it should be, and not in the additional in-patients admitted to treatment. Another satisfactory point in this connection is that since the year 1878 there have been no arrears of subscriptions. Although the income from these sources in 1891 amounted to £8,891, there were no subscriptions in arrear, as against an income of £2,029 in 1850, when the arrears amounted to £220. This is very creditable to all concerned, and reveals a sound system of finance and a due sense of duty on the part of the subscribers. There can be no doubt that the administration of the Leeds Infirmary is so intelligent and admirable that the people of Leeds will be well advised to place at the disposal of the managers all the money which they may from time to time declare to be necessary to enable them to maintain the present high state of efficiency to which the institution has attained.

#### The Out-Patient Department.

This department is very remarkable, seeing that it is the largest, and on the whole the most complete in the world. It consists of an immense central hall, round which are arranged the rooms for the medical staff and dispensers. It is administered on the numbers system, which supplies each patient with a number when he first applies, which afterwards represents a cloth case containing the prescription paper and the history of his case and treatment. When a patient arrives he is seen by the enquiry clerk, who gives him his prescription paper case on producing his number. This case he takes with him into the consulting-room, and after the doctor has done with it he takes it to the dispenser, where he gives it up in exchange for the medicine. This system, which has been long in force at the London and other hospitals, secures the preservation of the clinical material afforded by the out-patient department, and at the same time keeps the prescription papers and history forms in a condition to enable them to be bound up and preserved. In connection with the out-patient department there is an isolation-room opening into the street, to which all suspicious cases are at once removed. After a diagnosis has been made it is easy to communicate with the health authorities, and to secure the removal, when necessary, of all infectious cases to the isolation hospital. Each and all of the consulting rooms communicate with each other, so that it is easy for the staff to consult in difficult cases without unduly disturbing the patients. The fittings and arrangement of these consulting-rooms are wonderfully complete and up to date. The most noteworthy of them is the division assigned to the ophthalmic surgeon and his assistants, which constitutes, probably, the most complete and excellent ophthalmic clinic to be met with in this country. No one can visit this out-patient department without being impressed with the enormous developments that have taken place in medical science during the last quarter of a century in this country, nor will they fail to note that here the poorest citizen has advantages, when ill, which the richest cannot secure to anything like the same extent, no matter how much he is prepared to pay for such necessary aids to recovery.

There is another side, however, to the out-patients' question, which is forced upon the mind on visiting an enormous department like that recently opened at Leeds. Where is out-patient treatment to stop? Is every member of the community who prefers the hospital to the private consulting-room, to be at liberty to avail himself of the wonderful facilities afforded by the development of hospital buildings, such as those we have described? We would commend the following figures to the attention of (1) the authorities, and (2) the inhabitants of Leeds, in connection with this question. In 1850 the Leeds Infirmary treated only 3,217 out-patients, including those in-patients who were made out-patients. The numbers increased each decade as follows: In 1860 they were 4,712, an increase of 33 per cent.; in 1870 they were 7,496, a further increase of 60 per cent.; in 1880 they were 15,725, a further increase of more than 100 per cent.; and in 1891 they numbered nearly 29,000, a further increase in the last decade of nearly 100 per cent., as compared with the number admitted to treatment at the end of the previous ten years. Now, if we compare the number of out-patients per thousand and the position of the principal cities and towns of this country, we find that all the institutions of Leeds, numbering four hospitals and one dispensary, gave free medical relief to 182 persons out of every thousand of the population. These figures relate to three years ago, and they show that Leeds then stood thirteenth on the list of large towns in Great Britain and Ireland. In Dublin 459 persons out of every thousand of the population received free out-patient relief; in Liverpool, 382; in Edinburgh, 365; in Bristol, 347; in Leicester, 345; in Birmingham, 336; and in Newcastle, 289. Now, the problem which the authorities of the Leeds Infirmary and the inhabitants of Leeds will have to solve is, are they going to continue to occupy the relatively sound position they at present hold when compared with other large towns, on this out-patient question, or is the opening of the splendid new buildings and out-patient department (than which nothing better can be provided) to be the commencement of a new departure which will result in causing many thousands more inhabitants of Leeds to seek free out-patient relief than ever before? On the other hand, we admit, of course, that every well-administered hospital, so long as it undertakes the cure of the sick, must provide the very best accommodation and means to that end. We therefore heartily congratulate and thank the committee and medical staff of the Leeds Infirmary for the ability and knowledge displayed in the arrangement of the new buildings, which ought to make Leeds very proud indeed of the great hospital which so ably provides for the needs of the suffering poor.

### The Horse Ambulance.

The Leeds General Infirmary has provided for some years a horse ambulance, which is sent with a doctor and attendants, on being summoned by telephone. The cost of this ambulance, which has lessened the sufferings of many poor people during a number of years, is about £200 per annum, including repairs. It necessarily proves of great service for the removal of severe accidents with rapidity, so that freedom from great suffering and distress is attained. It was called into requisition over 500 times during 1891. This ambulance, however, is unfortunately subject to abuse, more from want of thought and judgment than from any other cause. It is frequently sent for to convey cases of an obviously trifling character, and the Board have found it necessary to call the attention of the authorities at mills, workshops, and other places to this fact, and to impress upon them that the sole object of the ambulance is the conveyance to the hospital of cases, the removal of which could not otherwise be effected without risk of further injury. In any case, it is a new departure which is worthy of the consideration of all hospital committees, and we think that the example set by the Leeds Infirmary Committee might be wisely followed by many other similar institutions.

### The Resident Staff.

One thing impressed us very much at the Leeds Infirmary. It was the intelligence, the whole-hearted devotion to the work, and the cheerful self-denial which characterised each and all of the members of the resident staff with whom we came in contact.

The general manager, Mr. Blair, has for many years ably presided over the internal arrangements of the Infirmary; Miss Fisher is one of the best of lady superintendents, whose practical common sense must prove of special value in the administration of a great hospital like that at Leeds; Miss Rapson, the assistant superintendent of nurses, most ably aids in the work; whilst the sisters and nurses show an intelligence which it is a pleasure to recognise and welcome. The resident medical staff, of whom the senior, an old Guy's man, whom we were glad to find in that position, are warmly interested in their work. They take a genuine pride in everything that concerns the well-being of the Infirmary, and the fact that some of them have resided there for several years shows that Leeds affords an opening for the younger generation of medical men for useful, scientific, and general work which should make these resident medical appointments much sought after by the best students of the principal medical schools throughout the country.

